# Krankenhausaufenthalte von Pflegeheimbewohnern in der letzten Lebensphase: eine Analyse von Krankenkassenroutinedaten

**DOI:** 10.1007/s00391-020-01716-3

**Published:** 2020-03-17

**Authors:** Falk Hoffmann, Katharina Allers

**Affiliations:** Department für Versorgungsforschung, Carl von Ossietzky Universität Oldenburg, Ammerländer Heerstr. 140, 26129 Oldenburg, Deutschland

**Keywords:** Pflegeheim, Versorgung am Ende des Lebens, Krankenhaus, Geschlechterunterschiede, Versorgungsforschung, Nursing homes, End-of-life care, Hospital use, Sex differences, Health services research

## Abstract

**Hintergrund:**

Im internationalen Vergleich versterben Pflegeheimbewohner in Deutschland häufig im Krankenhaus. Daten zu längeren Zeiträumen vor dem Tod und zu regionalen Unterschieden fehlen.

**Ziel der Arbeit:**

Es werden Häufigkeiten von Krankenhausaufenthalten bei Pflegeheimbewohnern in verschiedenen Perioden vor dem Tod analysiert. Zudem werden Unterschiede nach Alter, Geschlecht, Pflegestufe, Demenz und Bundesländern untersucht.

**Material und Methoden:**

Wir verwendeten Daten einer großen Krankenkasse und schlossen Pflegeheimbewohner im Mindestalter von 65 Jahren ein, die zwischen dem 01.01.2010 und dem 31. 12.2014 verstarben. Outcome war mindestens ein Krankenhausaufenthalt nach Heimeintritt in verschiedenen Phasen des letzten Lebensjahres. Vertiefende Analysen wurden für die Zeiträume 0 (entspricht Versterben im Krankenhaus), 28 und 365 Tage vor Tod durchgeführt.

**Ergebnisse:**

Von den insgesamt 67.328 verstorbenen Bewohnern (mittleres Alter: 85,3 Jahre; 69,8 % weiblich), verstarben 29,5 % im Krankenhaus. In den letzten 28 bzw. 365 Tagen vor Tod hatten 51,5 % bzw. 74,3 % mindestens einen Krankenhausaufenthalt. Diese Werte waren in ostdeutschen Bundesländern höher. In allen Zeiträumen wurden Männer häufiger hospitalisiert. Bewohner mit höherer Pflegestufe wurden seltener stationär behandelt, besonders unmittelbar vor dem Tod. Demenz hatte keinen nennenswerten Einfluss auf die Hospitalisierungshäufigkeiten.

**Diskussion:**

Etwa die Hälfte der Pflegeheimbewohner wird im letzten Lebensmonat stationär behandelt, und ein Drittel verstirbt im Krankenhaus, was, international betrachtet, hoch ist. Dass wir keine Unterschiede bei Bewohnern mit und ohne Demenz fanden, widerspricht ebenso internationalen Befunden. Somit besteht erheblicher Handlungsbedarf, die palliative Versorgung von Pflegeheimbewohnern zu optimieren.

## Abstract (en)

**Background:**

Compared to the international literature a higher proportion of German nursing home residents (NHR) die in hospital. Data on longer periods before death and on regional differences are not available.

**Objective:**

The frequency of hospitalizations of NHR in different periods during their last year of life were investigated. Differences between age, sex, level of nursing care, dementia and federal states were also assessed.

**Material and methods:**

This study used data of a large German health insurance fund and included NHR aged 65+ years who died between 1 January 2010 and 31 December 2014. We assessed the proportion of NHR with at least one hospitalization during different periods before death. In-depth analyses were conducted for 0 (which corresponds to in-hospital death), 28 and 365 days before death.

**Results:**

Of the 67,328 deceased residents (mean age: 85.3 years, 69.8% female), 29.5% died in hospital. A total of 51.5% and 74.3% were hospitalized during the last 28 and 365 days of life, respectively. These values were higher in the eastern parts of Germany. Males were hospitalized more often than women in all time periods. A higher care dependency was associated with fewer hospitalizations, especially shortly before death. There was no noticeable difference in the frequency of hospitalization between NHR with and without dementia.

**Conclusion:**

Approximately half of all NHR in Germany are hospitalized during the last month of life and one third die in hospital, which is relatively high compared to the international literature. No major differences were found between NHR with and without dementia, which is also contradictory to international studies. Overall, there is a need to optimize palliative care for NHR in Germany.

## Einleitung

Aktuell leben in Deutschland etwa 800.000 Menschen in Pflegeheimen; diese Zahl ist über die letzten Jahre angestiegen und wird dies im Zuge des demografischen Wandels auch weiter tun [39, 44]. International und national nimmt gleichzeitig die Bedeutung des Settings Pflegeheim für die Versorgung am Ende des Lebens zu [8, 40].

Pflegeheimbewohner sind neben einem hohen Alter durch eine Vielzahl chronischer Erkrankungen sowie körperliche und kognitive Einschränkungen gekennzeichnet [10, 36, 37]. Etwa die Hälfte der Bewohner leidet an einer Demenz [18, 22, 42]. Diese Bewohner unterscheiden sich in zahlreichen Aspekten von denen ohne Demenz. So sind Bewohner mit Demenz in der Regel älter [1, 25] und haben nach Heimeintritt eine niedrigere Sterblichkeit [13, 28]. Andererseits benötigen sie oftmals mehr Unterstützung als Bewohner ohne Demenz [25, 31, 43]. Bewohner mit Demenz versterben typischerweise an Komplikationen der Erkrankung [32, 43], wodurch sich auch der Versorgungsbedarf am Ende des Lebens von Bewohnern ohne Demenz unterscheidet.

Insgesamt sind die Hospitalisierungsraten von Bewohnern sowohl zum Zeitpunkt des Heimeintritts als auch in unmittelbarer Nähe zum Tod am höchsten und steigen insbesondere im letzten Lebensjahr deutlich an [2, 5, 9, 12, 16]. In einem kürzlich von uns durchgeführten systematischen Review (*n* = 35 Studien) fanden wir, dass im Median über alle Studien 22,6 % der Bewohner im Krankenhaus verstarben und im letzten Lebensmonat im Median 33,2 % hospitalisiert wurden [3]. Zu anderen Perioden innerhalb des letzten Monats vor dem Tod gab es lediglich zwei Studien. Insgesamt vergleichen auch nur wenige Studien das Versorgungsgeschehen von Bewohnern mit und ohne Demenz [24, 25, 43, 45]. Zudem zeigten sich international deutliche Unterschiede in Bezug auf Hospitalisierungen von Pflegeheimbewohnern am Lebensende. Die Mehrzahl der eingeschlossenen Studien kam aus den USA; aus Deutschland konnten wir lediglich eine ältere und eine eigene Studie einschließen. Diese legen nahe, dass in Deutschland ein vergleichsweise hoher Anteil der Pflegeheimbewohner unmittelbar vor dem Tod im Krankenhaus behandelt wird [2, 17, 35].

Ziel dieser Arbeit war es deshalb, in Ergänzung der bereits von uns publizierten Daten zum Versterben im Krankenhaus [2, 17] vertiefende Analysen zur Häufigkeit von Krankenhausaufenthalten bei Pflegeheimbewohnern in verschiedenen Perioden innerhalb des letzten Lebensjahres durchzuführen. Dabei sollten auch Unterschiede nach Alter, Geschlecht, Pflegestufe, Demenz und Bundesländern untersucht werden.

## Methodik

### Datenbasis, Studienpopulation und Outcome

Wir verwendeten Routinedaten der DAK-Gesundheit, die mit etwa 6 Mio. Versicherten bundesweit eine der größten gesetzlichen Krankenkassen ist. Die Studienpopulation setzte sich aus allen Versicherten im Mindestalter von 65 Jahren zusammen, die zwischen dem 01.01.2010 und dem 31.12.2014 erstmalig in ein Pflegeheim kamen. Dazu war es erforderlich, dass in den 365 Tagen vor Heimeintritt keine Leistungen vollstationärer Pflege abgerechnet wurden und die Personen in dieser Zeit durchgängig versichert waren. Für diese Studie wurden aus dieser Population nur Personen berücksichtigt, die nach Pflegeheimeintritt zwischen dem 01.01.2010 und dem 31.12.2014 verstarben.

Informationen zur vollstationären Pflege sowie zu Pflegestufen stammen aus den Routinedaten der gesetzlichen Pflegeversicherung. Die heute geltenden neuen Pflegegrade wurden erst 2017 eingeführt, somit liegen für alle Jahre Pflegestufen vor. Für alle Analysen wurde die Pflegestufe zum Todeszeitpunkt berücksichtigt.

Auf Basis ambulanter Diagnosen wurde identifiziert, ob die Bewohner an Demenz erkrankt waren. Analog zu früheren Studien [2, 19, 23], wurden dazu die folgenden ICD-10-Codes verwendet: F00.x, F01.x, F02.0, F02.3, F03, G30.x, G31.0, G31.1, G31.82, G31.9 und R54. Mindestens eine der entsprechenden Diagnosen musste im Quartal des Heimeintritts vorliegen.

Unser Outcome war mindestens ein Krankenhausaufenthalt nach Heimeintritt in verschiedenen Phasen vor dem Tod. Die berücksichtigten Phasen waren 0 Tage (entspricht dem Versterben im Krankenhaus), 3, 7, 14, 28, 90, 180 und 365 Tage vor Tod.

### Statistische Analyse

Nach der deskriptiven Darstellung der Baseline-Charakteristika wurde der Anteil der Bewohner mit mindestens einem Krankenhausaufenthalt in den 8 verschiedenen Zeiträumen ermittelt. Nenner waren in den Analysen jeweils alle im Pflegeheim verstorbenen Bewohner (wenn z. B. ein Bewohner bereits nach 30 Tagen Aufenthalt im Heim verstarb, wurden Krankenhausaufenthalte auch ausschließlich in dieser Phase in allen Analysen berücksichtigt). Der Anteil der Bewohner mit mindestens einem Krankenhausaufenthalt wurde für die gesamte Population sowie stratifiziert nach Alter zum Zeitpunkt des Todes (65–74, 75–84, 85–94 und 95+ Jahre), Geschlecht (Männer, Frauen), Pflegestufe zum Zeitpunkt des Todes (keine/I, II und III) sowie Demenz (ja, nein) ausgewertet.

Weitere vertiefende Analysen wurden für die Zeiträume 0, 28 und 365 Tage vor Tod durchgeführt. Hierzu zählte jeweils eine multiple logistische Regression, in die die oben genannten Variablen Alter (4 Kategorien), Geschlecht (2 Kategorien), Pflegestufe (3 Kategorien) und Demenz (2 Kategorien) einflossen und bei der zusätzlich für die Dauer des Heimaufenthaltes (4 Kategorien, unterteilt nach Quartilen) adjustiert wurde. Es wurden Odds Ratios (OR) mit entsprechenden 95 %-Konfidenzintervallen (95 %-KI) geschätzt.

Weiterhin wurden für diese 3 Zeiträume auch regionale Analysen nach Bundesländern durchgeführt, bei denen auf die Alters- und Geschlechterverteilung aller verstorbenen Pflegeheimbewohner standardisiert wurde. Zudem wurden die Hauptentlassungsdiagnosen aller Krankenhausaufenthalte in diesen 3 Zeiträumen ausgewertet. Diagnosen wurden nach den 12 verschiedenen von Ramroth et al. vorgeschlagenen Krankheitsgruppen klassifiziert [34, 35].

Alle statistischen Analysen wurden mit SAS für Windows Version 9.4 (SAS Institute Inc, Cary, NC, USA) durchgeführt.

## Ergebnisse

### Baseline-Charakteristika

Von den 127.227 zwischen 2010 und 2014 neu ins Pflegeheim aufgenommenen Versicherten verstarben in diesem Zeitraum insgesamt 67.328 Bewohner (52,9 %). Diese waren durchschnittlich 85,3 Jahre alt (Median: 86), mehr als zwei Drittel waren weiblichen Geschlechts (69,8 %), und 43,1 % wiesen Demenzdiagnosen auf. Zum Zeitpunkt des Todes waren 46,8 % in Pflegestufe II eingruppiert. Die Bewohner waren durchschnittlich 343,5 Tage im Heim, bevor sie verstarben. Die Verteilung ist jedoch mit einem Median von 189 Tagen rechtsschief. Insgesamt 17,4 % der Verstorbenen waren zum Zeitpunkt des Todes maximal 28 Tage und 64,6 % maximal 365 Tage im Heim. Die Anzahl verstorbener Bewohner schwankte je Bundesland zwischen 671 in Bremen und 13.388 in Nordrhein-Westfalen.

### Versterben im Krankenhaus

Von den 67.328 Bewohnern verstarben 19.887 im Krankenhaus (29,5 %) (Tab. 1). Der Anteil im Krankenhaus verstorbener Bewohner variierte mit dem Alter, jedoch ohne klaren Trend. In den 3 Altersgruppen zwischen 65 und 94 Jahren fanden sich Anteile zwischen 27,5 und 32,1 %, bei Personen im Mindestalter von 95 Jahren zeigte sich ein niedrigerer Wert (21,7 %). Im Vergleich zu Männern (32,4 %) verstarben weibliche Bewohner (28,3 %) seltener im Krankenhaus, während das Vorliegen einer Demenz keinen Einfluss hatte. Einen linearen Einfluss hatte die Pflegestufe, wobei mit zunehmendem Pflegebedarf weniger Bewohner im Krankenhaus verstarben.

**Table 1:** 

Anteil der Bewohner mit Hospitalisierungen vor Tod im Zeitraum von	0 Tagen (%)	3 Tagen (%)	7 Tagen (%)	14 Tagen (%)	28 Tagen (%)	90 Tagen (%)	180 Tagen (%)	365 Tagen (%)
*Alter*								
65–74 Jahre	27,5	30,5	35,7	42,3	50,2	60,7	64,3	66,7
75–84 Jahre	32,1	35,2	40,0	46,5	54,6	66,9	72,1	75,9
85–94 Jahre	29,6	32,5	37,2	43,6	51,6	64,6	70,8	75,9
95+ Jahre	21,7	24,2	28,6	34,2	40,4	52,3	58,8	66,7
*Geschlecht*								
Männer	32,4	35,9	41,3	47,3	55,8	67,7	72,9	76,7
Frauen	28,3	30,9	35,5	41,2	49,6	62,3	68,2	73,2
*Pflegestufe*								
Keine/I	41,3	44,5	49,5	55,5	62,5	71,2	74,9	77,4
II	29,0	32,0	37,0	43,7	51,8	64,7	70,4	74,6
III	17,5	19,7	23,9	29,9	38,6	53,7	62,3	70,1
*Demenz*								
Ja	29,2	32,0	36,8	43,2	51,3	64,7	71,2	76,8
Nein	29,8	32,8	37,6	43,9	51,6	63,4	68,5	72,4
*Gesamt*	29,5	32,4	37,2	43,6	51,5	63,9	69,6	74,3

Regional betrachtet variierte der Anteil im Krankenhaus verstorbener Bewohner zwischen 24,3 % in Baden-Württemberg und 34,6 % in Mecklenburg-Vorpommern (Tab. 2). Obwohl es insgesamt keine klaren Nord-Süd- oder Ost-West-Gefälle gab, lagen alle 6 ostdeutschen Bundesländer über dem Bundesdurchschnitt, wenn auch die Unterschiede teils sehr gering waren.

**Table 2:** 

Bundesland	Anteil der Bewohner mit Hospitalisierungen vor Tod im Zeitraum von		
	0 Tagen (%)	28 Tagen (%)	365 Tagen (%)
Baden-Württemberg	24,3	47,4	72,6
Bremen	26,6	46,2	71,4
Schleswig-Holstein	27,4	48,3	69,5
Hessen	29,2	51,8	75,3
Niedersachsen	29,3	50,6	73,7
Saarland	29,4	50,7	73,9
Thüringen	30,4	55,8	80,7
Nordrhein-Westfalen	30,7	50,8	73,0
Berlin	31,0	55,2	75,5
Bayern	31,6	54,6	77,5
Sachsen	31,6	55,7	78,6
Hamburg	31,8	53,6	73,5
Rheinland-Pfalz	32,4	54,1	75,4
Brandenburg	32,4	55,0	77,3
Sachsen-Anhalt	33,0	55,4	76,9
Mecklenburg-Vorpommern	34,6	58,9	78,7

### Hospitalisierung im Verlauf des letzten Jahres vor Tod

Der Anteil der Bewohner, die mindestens einmal stationär behandelt wurden, erhöht sich auf 51,5 % bzw. 63,9 %, wenn man die letzten 28 bzw. 90 Tage vor Tod betrachtet (Tab. 1). Bezogen auf das letzte Lebensjahr waren drei Viertel (74,3 %) mindestens einmal im Krankenhaus. Von den 50.002 Bewohnern, die im letzten Lebensjahr stationär behandelt wurden, hatten 23.934 (47,9 %) genau einen und 26.068 (52,1 %) mehrere Aufenthalte.

Die bereits beim Versterben im Krankenhaus festgestellten Unterschiede nach Alter, Geschlecht und Pflegestufe setzen sich auch bei Betrachtung verschiedener Phasen innerhalb des letzten Lebensjahres fort (Tab. 1). Allerdings werden die auch innerhalb der letzten 28 Tage vor dem Tod noch bestehenden großen Unterschied in Abhängigkeit von der Pflegestufe (keine/I; II und III: 62,5 %; 51,8 % und 38,6 %) bei Betrachtung des kompletten letzten Lebensjahrs deutlich kleiner (77,4 %; 74,6 % und 70,1 %).

Die multivariaten Analysen bestätigen diese Befunde weitgehend (Tab. 3). Der Einfluss des männlichen Geschlechts und des Alters bleibt weitgehend unverändert, während der Einfluss der Pflegestufe abnimmt, wenn die letzten 365 Tage betrachtet werden. Das Vorliegen einer Demenzdiagnose geht in allen Perioden mit einer geringfügig höheren Chance einher, hospitalisiert zu werden.

**Table 3:** 

	Hospitalisierungen vor Tod im Zeitraum von, OR (95 %-KI)		
	0 Tagen	28 Tagen	365 Tagen
*Alter*			
65–74 Jahre	1,50 (1,37–1,64)	1,41 (1,30–1,52)	1,36 (1,25–1,47)
75–84 Jahre	1,77 (1,64–1,91)	1,69 (1,58–1,80)	1,82 (1,70–1,96)
85–94 Jahre	1,49 (1,39–1,60)	1,51 (1,42–1,61)	1,64 (1,54–1,75)
95+ Jahre	1	1	1
*Geschlecht*			
Männer	1,24 (1,19–1,28)	1,27 (1,23–1,32)	1,24 (1,19–1,29)
Frauen	1	1	1
*Pflegestufe*			
Keine/I	3,59 (3,42–3,78)	2,69 (2,58–2,82)	1,70 (1,62‑1,79)
II	2,03 (1,94–2,13)	1,72 (1,65–1,78)	1,41 (1,35–1,47)
III	1	1	1
*Demenz*			
Ja	1,04 (1,002–1,08)	1,09 (1,06–1,13)	1,12 (1,08–1,16)
Nein	1	1	1
*Dauer im Pflegeheim*			
Q1 (≤47 Tage)	1	1	1
Q2 (48–189 Tage)	1,65 (1,57–1,73)	0,99 (0,94–1,03)	2,65 (2,52–2,78)
Q3 (190–540 Tage)	1,85 (1,76–1,95)	1,07 (1,02–1,11)	4,22 (4,00‑4,45)
Q4 (541–1819 Tage)	1,72 (1,63–1,81)	0,94 (0,90–0,99)	2,55 (2,43–2,68)

*OR* Odds Ratio; *95%-KI* 95% Konfidenzintervall

Die ebenso in Bezug auf Versterben im Krankenhaus gefundenen regionalen Unterschiede bilden sich auch bei der Betrachtung von Hospitalisierungen in den letzten 28 (Spanne zwischen 46,2 und 58,9 %) und 365 Tagen vor Tod (69,5–80,7 %) ab. Bei beiden Zeitspannen liegen jeweils die ostdeutschen Länder über dem Bundesdurchschnitt (Tab. 2).

Betrachtet man die Hauptentlassungsdiagnosen (Tab. 4), spielen Infektionen (22,4 %) bei den im Krankenhaus verstorbenen Bewohnern die größte Rolle, während deren Bedeutung bei allen Hospitalisierungen in den letzten 28 (18,6 %) und 365 Tagen (15,2 %) rückläufig ist. Gleiches gilt auch für kardiovaskuläre Erkrankungen. Auf der anderen Seite nehmen Verletzungen an Bedeutung zu.

**Table 4:** 

Hauptentlassungsdiagnosen (ICD-10)	Hospitalisierungen vor Tod im Zeitraum von		
	0 Tagen (%)	28 Tagen (%)	365 Tagen (%)
Infektionen (A00–B99, J10–J18, L00–L08, N30, N39)	22,4	18,6	15,2
Kardiovaskuläre Erkrankungen (D50–D89, I00–I59, I70–I99)	21,7	18,0	16,8
Erkrankungen des Verdauungssystems (K00–K93)	9,5	8,7	8,6
Atemwegserkrankungen (J00–J09, J19–J99)	9,4	8,0	6,4
Verletzungen (R55, S00–T98)	8,3	10,5	14,4
Andere Erkrankungen (nicht in den anderen Gruppen)	7,9	8,2	8,9
Krebserkrankungen (C00–D48)	6,5	8,9	8,1
Zerebrovaskuläre Erkrankungen (G45–G46, I60–I69)	6,4	7,1	5,7
Hormon‑, Ernährungs‑, Stoffwechselerkrankungen (E00–E90, N18–N19)	4,2	5,6	6,4
Erkrankungen des Nerven- und Sinnessystems (G00–G44, G47–G99)	1,9	2,8	3,6
Psychiatrische Erkrankungen (F00–F99)	1,1	2,5	4,0
Erkrankungen des Muskelsystems (M00–M99)	0,7	1,1	1,8

## Diskussion

### Vergleich der Ergebnisse mit der Literatur

Insgesamt wird etwa die Hälfte der Pflegeheimbewohner im letzten Lebensmonat stationär im Krankenhaus behandelt, und etwa ein Drittel verstirbt dort. Etwa drei Viertel waren im letzten halben bzw. letzten Jahr vor Tod im Krankenhaus. Somit liegt die vulnerable Phase v. a. im letzten Lebensmonat. Männer wurden in allen Perioden häufiger stationär behandelt als Frauen, und es zeigten sich ein Einfluss der Pflegestufe sowie regionale Unterschiede. Demenz hat jedoch keinen nennenswerten Einfluss auf Hospitalisierungen im letzten Lebensjahr von Pflegeheimbewohnern.

International existieren erhebliche Unterschiede in Bezug auf Hospitalisierungen von Pflegeheimbewohnern am Lebensende. In einem kürzlich von uns durchgeführten systematischen Review lag der Median beim Anteil im Krankenhaus verstorbener Bewohner (*n* = 29 Studien) bei 22,6 % und für Krankenhausaufenthalte im letzten Lebensmonat bei 33,2 % (*n* = 12) [3]. Es ist somit klar ersichtlich, dass Deutschland mit entsprechenden Anteilen von 29,5 % bzw. 51,5 % deutlich höher liegt. Mit 28,9 % im Krankenhaus verstorbener Bewohner wurden nahezu identische Werte bereits in einer vor über 15 Jahren in Süddeutschland durchgeführten Studie gefunden [35]. Dies deutet darauf hin, dass es hierbei seitdem kaum Veränderungen gab.

Einen großen Einfluss auf Hospitalisierungen in den letzten Wochen vor Tod hatte die Pflegestufe. Dies deckt sich mit anderen internationalen Studien, die zeigten, dass Bewohner mit höherem Pflegebedarf seltener im Krankenhaus versterben [29, 30]. Dieser Effekt wird geringer, wenn man längere Phasen vor Tod betrachtet. Dies könnte möglicherweise dadurch bedingt sein, dass ausschließlich die Pflegestufe zum Zeitpunkt des Todes berücksichtigt wurde. Andererseits legen frühere Analysen nahe, dass die Pflegestufe auf Hospitalisierungen im Jahr vor als auch nach Heimeintritt kaum einen Einfluss hat [16]. Somit scheint es wesentlich plausibler, dass die Pflegestufe insbesondere einen Einfluss auf die Wahrscheinlichkeit von Hospitalisierungen unmittelbar am Lebensende hat. Die Tatsache, dass kurz vor Tod Infektionen als Diagnosen stationärer Aufenthalte eine wichtigere Rolle spielen und Verletzungen anteilig weniger relevant sind, wurde bereits mit älteren Daten von Ramroth et al. festgestellt [33].

Über alle betrachteten Perioden vor Tod wurden männliche Bewohner häufiger im Krankenhaus behandelt als weibliche. Dies deckt sich mit Befunden aus 2 systematischen Reviews unserer Arbeitsgruppe, einerseits zu Hospitalisierungen am Lebensende [3] sowie zu Hospitalisierungen insgesamt nach Heimeintritt [15]. Dass solche Unterschiede in nahezu allen internationalen Studien gefunden wurden, erhärtet die Evidenz. Bisherige Untersuchungen von Todesbescheinigungen in der Gesamtbevölkerung sowie begrenzt auf Patienten mit Demenz fanden ebenfalls einen deutlich höheren Anteil an Männern, deren Sterbeort das Krankenhaus ist, während Frauen häufiger im Pflegeheim sterben [7, 8]. In den entsprechenden Studien wird dies allerdings oftmals damit erklärt, dass Männer aufgrund der geringeren Lebenserwartung eine höhere Wahrscheinlichkeit dafür haben, vom Partner überlebt zu werden. Dadurch würden Männer am Lebensende häufiger von Angehörigen im häuslichen Umfeld betreut, während Frauen dann allein leben und deshalb im Heim versorgt werden. Auch wenn dieser Erklärungsansatz naheliegt, erschließt sich damit nicht, wieso männliche Pflegeheimbewohner häufiger im Krankenhaus sterben. Stattdessen scheint es sich hierbei um einen echten Geschlechtereffekt zu handeln. Obwohl Fragen zu geschlechtsspezifischen Unterschieden in den letzten Jahren zunehmend an Relevanz gewinnen, gibt es für das Phänomen, dass männliche Pflegeheimbewohner insgesamt und auch in der letzten Lebensphase häufiger im Krankenhaus behandelt werden, bisher keine Erklärung.

Neben dem klaren und über alle Perioden konstanten Geschlechterunterschied, sehen die Ergebnisse in Bezug auf den Einfluss des Alters weniger deutlich aus. Häufig werden in der Literatur auch verschiedene Altersgruppen verwendet, was eine Interpretation erschwert [15]. Möglicherweise unterscheidet sich der Einfluss von Alter auch zwischen Bewohnern mit und ohne Demenz [17] sowie zwischen unterschiedlichen Perioden des Heimaufenthalts [16].

Wir fanden in allen Perioden keine nennenswerten Unterschiede in der Hospitalisierungshäufigkeit zwischen Bewohnern mit und ohne Demenz. Dies ist ein unerwarteter Befund, denn laut dem kürzlich von uns durchgeführten systematischen Review finden sämtliche nicht aus Deutschland kommenden Studien, welche Hospitalisierungen in den letzten 4 Lebenswochen von Pflegeheimbewohnern mit und ohne Demenz vergleichen, niedrigere Hospitalisierungsraten bei Vorliegen einer Demenz [6, 24, 25, 43, 45]. Teils zeigten diese Studien sogar erhebliche Unterschiede zwischen den Gruppen, wie beispielsweise Krishnan et al. (0 % mit bzw. 11,7 % ohne Demenz) [24] oder Sloane et al. (6,9 % bzw. 13,8 %) beim Versterben im Krankenhaus [43]. Im Vergleich dazu lagen die Anteile in unserer Studie bei 29,2 % (Demenz) bzw. 29,8 % (Nicht-Demenz). Gerade Bewohner mit Demenz profitieren am Ende des Lebens nicht von einer deutlich aggressiveren Therapie im Krankenhaus, da diese den Verlauf der Erkrankung nicht positiv beeinflusst [11, 21]. Diese Erkenntnis hat sich offenbar noch nicht in der deutschen Versorgungslandschaft durchgesetzt. Gozalo et al. haben jegliche Hospitalisierungen bei Bewohnern mit Demenz in den letzten 3 Lebenstagen als belastend („burdensome“) definiert [11]. In unserer Studie war die Aufenthaltsdauer bei 32,6 % der Bewohner, die im Krankenhaus verstarben, maximal 3 Tage (33,3 % mit bzw. 32,2 % ohne Demenz). Dies unterstreicht noch einmal den deutlichen Handlungsbedarf in Deutschland.

Eine mögliche und wichtige Maßnahme zur Reduktion von Krankenhausaufenthalten am Lebensende ist die Stärkung der Palliativversorgung in deutschen Pflegeheimen. Laut einer niederländischen Studie lag der Anteil der Krankenhauseinweisungen im letzten Lebensmonat von Pflegeheimbewohnern mit Demenz bei lediglich 8 %, während für die Mehrheit der Pflegeheimbewohner kurz vor dem Tod ein palliatives Versorgungsziel im Vordergrund stand und belastende kurative Maßnahmen vermieden wurden [14]. Darüber hinaus stellt Advance Care Planning, die vorausschauende Versorgungsplanung mit dem Ziel, die Wünsche einer Person hinsichtlich zukünftiger medizinischen Behandlungsentscheidungen und Versorgung im Falle der Nichteinwilligungsfähigkeit zu besprechen und zu dokumentieren [38], eine wichtige Grundlage dar, um vermeidbare und unerwünschte Krankenhausaufenthalte zu reduzieren [4]. So zeigt auch eine Studie aus Belgien, dass Pflegeheimbewohner mit Demenz ohne Patientenverfügung im letzten Lebensmonat häufiger ins Krankenhaus kamen als jene mit einer Verfügung [21]. Insbesondere bei Personen mit Demenz ist es wichtig, dass diese Gespräche zu einem möglichst frühen Zeitpunkt beginnen, so lange die Personen sich noch zu ihren Einstellungen und Wünschen äußern können.

### Stärken und Schwächen

Wesentliche Stärke dieser Arbeit ist die große Stichprobe von mehr als 67.000 verstorbenen Pflegeheimbewohnern, die sich über das komplette Bundesgebiet verteilen. Wir haben für alle Analysen den gleichen Nenner gewählt und somit z. B. für die letzten 365 Tage vor Tod ebenfalls alle 67.328 Bewohner eingeschlossen, obwohl lediglich 23.855 diese komplette Zeit im Heim verbracht haben und über die komplette Periode unter Risiko für Krankenhausaufenthalte standen. Würde man jedoch ausschließlich diese Bewohner berücksichtigen, schließt man systematisch morbidere Bewohner mit einer schnelleren Zustandsverschlechterung aus und erzeugt somit eine künstliche Population. In den Regressionsanalysen haben wir jedoch zusätzlich für die Dauer des Heimaufenthalts adjustiert.

Demenzdiagnosen wurden ausschließlich im Quartal des Heimeintritts betrachtet, und die damit gefundene Demenzprävalenz von 43 % ist niedriger als in der Literatur [18, 22, 42]. Ein systematischer Review fand im Median über alle berücksichtigten Studien eine Demenzprävalenz von 58 % bei Pflegeheimbewohnern [42]. Demenz ist ein wesentlicher Grund für den Eintritt ins Pflegeheim [26, 41], trotzdem könnten Diagnosen erst zu einem späteren Zeitpunkt abgerechnet werden. Berücksichtigt man jegliche Zeit nach dem Heimeintritt, erhöht sich die Prävalenz einer Demenz auf 56 %, allerdings bleibt der Anteil im Krankenhaus verstorbener Bewohner unverändert (29,4 und 29,8 % bei Bewohnern mit und ohne Demenz) [17]. Ebenso zeigen sich keine Unterschiede für die letzten 28 Tage (51,1 und 52,0 % bei Bewohnern mit und ohne Demenz). Dies untermauert die Robustheit unserer Ergebnisse in Bezug auf mögliche Unterschiede zwischen Bewohnern mit und ohne Demenz.

Routinedaten haben zur Untersuchung dieser Forschungsfrage erhebliche Vorteile, da sich mit ihnen für die vulnerable Gruppe von Pflegeheimbewohnern auch retrospektiv nach dem Tod Hospitalisierungen in der letzten Lebensphase valide abbilden lassen. Allerdings fehlen in diesen Daten v. a. wichtige klinische Angaben, beispielsweise zu kognitiven Einschränkungen, oder inwieweit eine Patientenverfügung vorliegt und was deren Inhalt ist. Auch Faktoren auf Seiten der Heime, die einen relevanten Einfluss haben [46], wie beispielsweise der Pflegeschlüssel oder Qualifikation des Personals, konnten nicht berücksichtigt werden.

Zudem wurden ausschließlich Daten einer einzelnen Krankenkasse verwendet, und es ist bekannt, dass die DAK-Gesundheit einen höheren Anteil an Personen mit chronischen Erkrankungen versichert [20]. Nichtsdestotrotz konnte gezeigt werden, dass die Hospitalisierungsraten in der DAK-Gesundheit denen der deutschen Gesamtbevölkerung entsprechen [27]. Trotzdem dürfen Ergebnisse von Studien einer einzelnen Kasse nicht unkritisch auf die Gesamtbevölkerung extrapoliert werden.

## Fazit

- In Deutschland wird etwa die Hälfte der Pflegeheimbewohner im letzten Lebensmonat stationär behandelt, und ein Drittel verstirbt im Krankenhaus.
- Diese Anteile sind, international betrachtet, vergleichsweise hoch, zudem existieren regionale Unterschiede.
- Auffällig ist auch, dass Hospitalisierungen am Ende des Lebens bei Bewohnern mit und ohne Demenz nahezu gleich häufig vorkommen.
- Alle verfügbaren internationalen Studien finden hier teils deutlich niedrigere Anteile bei Demenz.
- Somit besteht erheblicher Handlungsbedarf, die palliative Versorgung von Pflegeheimbewohnern in Deutschland am Lebensende, insbesondere auch bei Demenz, zu optimieren.
